# Changes in the Structure of the Neuromuscular Junction and Muscle Fiber Types Following an Acute Injury Model Induced by Eccentric Contraction

**DOI:** 10.3390/cimb48030325

**Published:** 2026-03-19

**Authors:** Mariana Baptista, Jurandyr Pimentel Neto, Matheus Bertanha Fior, Isabella Gomes, Adriano Polican Ciena

**Affiliations:** Laboratory of Morphology and Physical Activity (LAMAF), Institute of Biosciences, São Paulo State University, Rio Claro 13506900, São Paulo, Brazil; mariana.baptista@unesp.br (M.B.); jurandyr.pimentel@unesp.br (J.P.N.); matheus.b.fior@unesp.br (M.B.F.); i.gomes@unesp.br (I.G.)

**Keywords:** muscle injury, eccentric contraction, neuromuscular junction, presynaptic region, postsynaptic region

## Abstract

The neuromuscular junction (NMJ) is responsible for transmitting neural signals that trigger muscle contraction. Muscle injuries cause damage to cellular structures and trigger local inflammatory processes. In this context, eccentric contraction was used as an experimental model because it involves excessive stretching, generating mechanical stress. Twenty-five adult male Wistar rats were distributed into groups: Control (C) (*n* = 5) and Injury (I) (*n* = 20). The protocol was performed on a treadmill and consisted of 18 sets/5 min/16 m/min speed, with intervals, and with a negative incline (−16º). The analyses consisted of histochemical techniques, such as myofibrillar ATPase and immunofluorescence (calcium channels, synaptophysin and α-bungarotoxin). Group I-0H showed alterations in the presynaptic region and an increase in Type I fibers. I-24H presented disorganization in the postsynaptic region. In I-4D, we observed the reorganization of neuromuscular activity, while I-7D presented greater density and cross-sectional area (CSA) of Type II fibers. It is concluded that the protocol promotes changes in NMJ structure and fiber distribution, mainly in I-24H. In I-4d, a reorganization of neuromuscular activity is observed, and in I-7D, a structural indicator consistent with recovery demonstrates the skeletal muscle’s ability to adapt to injury.

## 1. Introduction

The neuromuscular junction (NMJ) is a key component of the nervous system that communicates with skeletal muscle tissue, contributing to muscle contraction [1]. The NMJ has three specific regions: the presynaptic region, the site preceding the transduction of the chemical signal; the synaptic cleft, the space for the transmission of nerve impulses between the motor axon terminal and the sarcolemma of the muscle fiber; and the postsynaptic membrane, formed by the sarcoplasmic membrane that surrounds the muscle fiber, composed of receptors for ACh (acetylcholine) molecules [2,3].

Neuromuscular integrity corresponds to the constant communication between the motor neuron and the muscle fiber. Development, repair, and tissue degeneration, as seen in conditions such as disuse muscle atrophy, aging, and denervation, can affect its functionality and that of other associated systems [4].

Some proteins associated with synaptic structures contribute to this integrity, such as synaptophysin, a structural glycoprotein that is one of the main components of synaptic vesicle membranes and that represents 7% of all membrane proteins. Thus, it is widely used as a presynaptic marker, since its presence indicates the density and integrity of nerve endings at the NMJ. Its detection also allows the evaluation of synaptic plasticity and remodeling processes under physiological or pathological conditions [5].

The interaction of vesicles with the plasma membrane occurs through the formation of fusion pores. In this context, calcium channels, located in the presynaptic membrane, are primarily responsible for the entry of calcium that triggers this fusion and results in the release of synaptic vesicles filled with acetylcholine (ACh). Once released into the synaptic cleft, ACh binds to nicotinic receptors present in the sarcolemma of the muscle fiber. This binding causes depolarization of the postsynaptic membrane, which initiates the action potential that gives rise to muscle contraction [6].

In this context the promotion of the NMJ structure can occur through the implementation of physical exercises demonstrated in experimental models [7,8], such as downhill treadmill running [9].

Skeletal striated muscle exhibits distinct contraction characteristics, being fast in Type II fibers and slow in Type I fibers, due to the overlap of actin and myosin filaments [10,11]. Slow-twitch fibers are primarily innervated by motor neurons, which generate low-frequency firings using predominantly aerobic energy, with tension maintained for long periods [12]. Fast-twitch fibers, in turn, are innervated by motor neurons that generate high-frequency firings, with high conduction velocity, and can maintain tension for short periods [13].

Despite the effectiveness of the neuromuscular system in regeneration, alterations in the NMJ can compromise the interaction between the peripheral nervous system and skeletal striated muscle, especially in injury situations [14]. In the sports context, muscle injuries have a high incidence, with emphasis on contusions and strains, including the injury induced by eccentric contraction [15].

During eccentric contraction, there is a simultaneous application of force and stretching on the muscle fibers, which increases the tension in these structures. For this reason, the eccentric contraction model has been widely used in experimental studies on muscle injuries [16].

The mechanical and metabolic stimulus induced by eccentric contraction may reflect the repercussions of the treadmill running protocol on neuromuscular recruitment. Furthermore, the possible morphometric adaptations of the pre- and post-synaptic structures may be directly related to the muscle contraction and fatigue observed from the different myofibrillar types.

## 2. Materials and Methods

### 2.1. Animals

We distributed twenty-five adult male Wistar rats (100 days old) (without sample loss) into two groups: Control (C) (*n* = 5), subjected to adaptation of the muscle injury protocol, and Injury (I) (*n* = 20), subjected to the eccentric contraction injury model (downhill) and subsequently divided for the analysis of the subgroups I-0H, I-24H, I-4D and I-7D (*n* = 5/subgroup). Sample size calculation was performed using the statistical program G*Power version 3.1, with a large effect size (f = 0.784), power of 80% (B = 0.05) and α = 0.05 [17].

The animals originated from the Central Animal Facility of UNESP—Botucatu Campus, SP, and were allocated to the Sectoral Animal Facility of the Anatomy Laboratory of the Department of Physical Education of the Institute of Biosciences of UNESP—Rio Claro/SP Campus. They were housed in collective cages and received standard balanced feed (Purina^®^) and water ad libitum, with an ambient temperature controlled at 23 °C ± 2 °C, with a light/dark photoperiod of 12 h. All procedures applied in this study were approved by the Ethics Committee on the Use of Animals (CEUA—nº 2223—25/2023) of the Institute of Biosciences of the São Paulo State University “Júlio de Mesquita Filho” Campus of Rio Claro—SP.

### 2.2. Induced Injury Protocol

Before the start of the protocol, all animals underwent an adaptation period. This process consisted of a brief treadmill running session, lasting five minutes, at a speed of 16 m/min and a negative incline of 16°.

The protocol consisted of a single eccentric exercise session, composed of 18 downhill treadmill running sets, at the same speed and incline as those in the adaptation period. Each set lasted five minutes, interspersed with two minutes of rest, totaling 2 h and 6 min of protocol, as described by Kanazawa et al. [16]. Throughout the procedure, the animals were constantly monitored by two experienced researchers who intervened only when necessary using a soft-bristled brush on the tail area to stimulate movement.

After the exercise, we moved the animals to a controlled environment, with free access to water and food, under the same conditions as the animals in Group C, until the time of sample collection. The experimental time points (groups) chosen were 0 h, 24 h, 4 days, and 7 days after the protocol (acute period) [18,19]. The body mass (g) of the animals was measured at the time of collection using a precision digital scale (TOMATE™ 440, Campinas, Brazil) with an average of 397.8 g.

### 2.3. Sample Processing

Animals from all experimental groups were euthanized by anesthetic overdose using Ketamine (300 mg/kg) and Xylazine (30 mg/kg), administered intraperitoneally. Subsequently, muscle samples from the triceps brachii were dissected bilaterally. The samples were subjected to cryofixation by immersion in isopentane cooled in liquid nitrogen (−196 °C). After this process, they were temporarily stored in a biofreezer (−80 °C) until histological sectioning was performed in a cryostat (HM 505 E, MICROM^®^, Walldorf, Germany).

### 2.4. ATPase Myofibrilar

We made serial 10 µm thick cross-sections of the middle third of the right triceps brachii muscle (HM 505E cryostat, MICROM^®^). For fiber type differentiation, the sections were processed to pH 4.3 and pre-incubated in a 0.1 M sodium acetate and 10 mM EDTA buffer solution for 10 min at 4 °C. The slides from each experimental group (*n* = 20/group) were incubated for 30 min at 37 °C with a solution containing 15 mg of ATP, 15 mL of glycine/NaCl/CaCl^2^ buffer, and 46.5 mg of dithiothreitol (DTT). Then, the slides were washed with distilled water, incubated with 2% cobalt chloride for 7 min, washed again with distilled water, dehydrated in a series of alcohols (70%, 90%, 95% and 100%), finished with xylene and mounted with Entellan^®^ (Darmstadt, Germany - Sigma-Aldrich) [20].

For morphometric analyses, multiple fibers were measured per animal, and the mean value for each animal was used for statistical analysis, considering the animal as the experimental unit. The images (*n* = 15 images/animal/group) were obtained at 100× magnification using a light microscope (Carl Zeiss Microimaging, Axioskop 40, Göttingen, Germany) at the Laboratory of Morphology and Physical Activity (LAMAF) of the Institute of Biosciences at São Paulo State University (UNESP), Rio Claro/SP Campus. Based on ATPase staining, it was possible to differentiate the muscle fiber phenotypes into types I and II, for subsequent measurement of the cross-sectional area: CSA (mm^2^) (*n* = 600/fiber type/group) and volume of the muscle fiber types (un), both measured using ImageJ^®^ 1.54p software (NIH, Bethesda, MD, USA).

### 2.5. Immunofluorescence

Five animals from each experimental group were used, with samples taken from the distal third of the left triceps brachii muscle. The samples were used to visualize the pre- and post-synaptic components of each neuromuscular junction (NMJ), where longitudinal sections (20 µm) (HM 505 E cryostat, MICROM^®^) were collected on silanized histological slides (*n* = 10/group/antibody). The sections were washed twice for 5 min with phosphate-buffered saline (PBS), permeabilized with 0.1% Triton X-100 diluted in PBS, and blocked with 1% bovine serum albumin (BSA) and 5% normal goat serum (NGS) diluted in PBS.

#### 2.5.1. Presynaptic and Postsynaptic Region

After initial processing, the slides (*n* = 10/group) were incubated overnight for 16 h with a solution containing the primary antibody against calcium channels (1:500), diluted in PBS with 1% BSA. After incubation, the samples were washed twice with PBS for 5 min each time. Then, the secondary antibody Alexa Fluor 488 (anti-rabbit, 1:1000) was applied for 1 h. After this process, the slides were mounted with Prolong^®^ Antifade (Molecular Probes, Eugene, OR, USA) and stored at −20 °C [21].

For identification of the postsynaptic region, samples (*n* = 10/group) were washed with PBS buffer solution and incubated overnight in a solution containing the primary antibodies α-bungarotoxin conjugated with synaptophysin conjugate (presynaptic region) (BTX; Molecular Probes^®^, Eugene, OR, USA). Subsequently, the slides were washed twice with PBS for 5 min, mounted with Prolong^®^ (Molecular Probes, Eugene, OR, USA), and stored at −20 °C.

#### 2.5.2. NMJ Morphometry

For neuromuscular junction morphometric analyses, multiple images of neuromuscular junctions were obtained from each animal. These images were treated as subsamples, and the mean value for each animal was calculated and used for statistical analysis, considering the animal as the experimental unit. The images (*n* = 15/animal/group/technique) were obtained using the Olympus^®^ BX61 fluorescence microscope at the Bacterial Genetics Laboratory (LGB), IB—UNESP Rio Claro/SP, with the 100× objective (1000× magnification) and 20× objective (200× magnification). Morphometrics of the pre- and post-synaptic regions of the NMJ were applied using manual and semi-automated methodologies, performed with the aid of ImageJ^®^ software (NIH, Bethesda, MD, USA), to obtain the following variables: Total area (μm^2^), stained area (μm^2^), total perimeter (μm), stained perimeter (μm^2^), diameter (μm), dispersion (%), number of clusters (u), cluster area (μm^2^) and fragmentation (0–2) [22,23].

#### 2.5.3. Statistical Analysis

After obtaining the data, outliers (ROUT, Q = 1%) were identified and normality analysis was established using the Shapiro-Wilk test, in addition to one-way ANOVA and two-way ANOVA with Bonferroni post-hoc test. A significance level of *p* < 0.05 was defined for the analyses, and all statistical tests were performed using GraphPad Prism 9.0.0^®^ software.

## 3. Results

### 3.1. ATPase Myofibrilar

**Figure 1:**
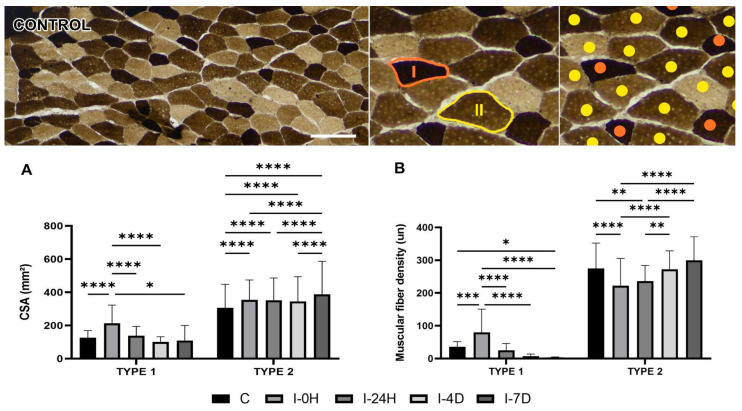
Data on cross-sectional area (CSA) and fiber density of type I (measured in orange) and type II (measured in yellow) groups (*n* = 5/group) from pH 4.3. Means and standard deviations of (**A**) CSA (mm^2^) and (**B**) fiber density (un) of all experimental groups. Data statistically treated using two-way ANOVA analysis with Bonferroni post-test with a significance level of *p* < 0.05. Bar: 1000 µm. Magnification: 100×. CSA type I: **** C≠I-0H (*p* < 0.0001), **** I-0H≠I-24H (*p* < 0.0001), **** I-0H≠I-4D (*p* < 0.0001), * I-0H≠I-7D (*p* = 0.0168); CSA type II: **** C≠I-0H (*p* < 0.0001), **** C≠I-24H (*p* < 0.0001), **** C≠I-4D (*p* < 0.0001), **** C≠I-7D (*p* < 0.0001), **** I-0H≠ I-7D (*p* < 0.0001), **** I-24H≠ I-7D (*p* < 0.0001), **** I-4D≠ I-7D (*p* < 0.0001); Type I density: *** C≠I-0H (*p* = 0.0008), * C≠I-7D (*p* = 0.0265), **** I-0H≠I-24H (*p* < 0.0001), **** I-0H≠I-4D (*p* < 0.0001), **** I-0H≠I-7D (*p* < 0.0001); Type II density: **** C≠I-0H (*p* < 0.0001), ** C≠I-24H (*p* = 0.0015), **** I-0H≠I-4D (*p* < 0.0001), **** I-0H≠I-7D (*p* < 0.0001), ** I-24H≠I-4D (*p* = 0.0033), **** I-24H≠I-7D (*p* < 0.0001).

When measuring the cross-sectional area of type I fiber, an increase of 69.25% and 9.54% was observed in groups I-0H (*p* < 0.0001) and I-24H, and there was a reduction of 19.81% in I-4D and 12.77% in I-7D, in relation to the Control. In type II fiber, there was an increase of 16.03%, 15.02%, 12.93% and 26.92% in groups I-0H (*p* < 0.0001), I-24H (*p* < 0.0001), I-4D (*p* < 0.0001), and I-7D (*p* < 0.0001), respectively (Figure 1A).

The density of type I fiber, in turn, was 122.65% higher in I-0H (*p* = 0.0008) and 29.59%, 78.68%, and 88.84% lower in I-24H, I-4D, and I-7D (*p* = 0.0265), respectively, compared to C. The density of type II fiber was 19.07% lower in I-0H (*p* < 0.0001), 14.05% lower in I-24H (0.0015), 0.76% lower in I-4D, and 8.99% higher in I-7D (Figure 1B).

### 3.2. Presynaptic Region: Synaptophysin

**Figure 2:**
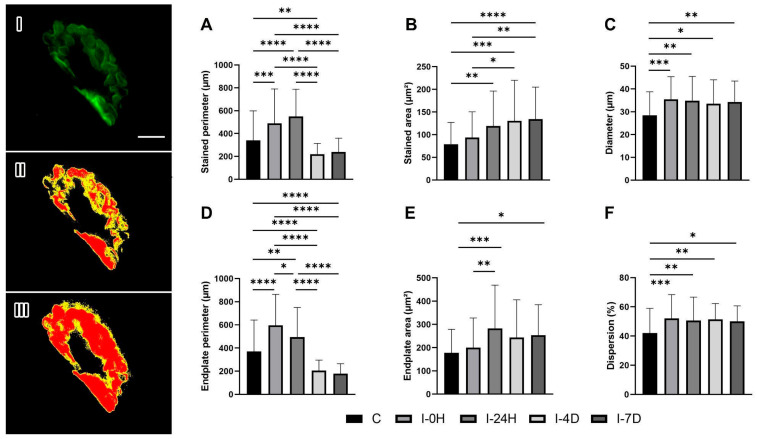
Synaptophysin (green staining) immunofluorescence microscopy (**I**) and measurement of stained area and perimeter (**II**) and total area and perimeter (**III**). Means and standard deviations of (**A**) stained perimeter (µm), (**B**) stained area (µm^2^), (**C**) diameter (µm), (**D**) endplate perimeter (µm), (**E**) endplate area (µm^2^) and (**F**) dispersion (%) of experimental groups (*n* = 5/group). Data statistically treated using one-way ANOVA analysis with Bonferroni post-hoc test with a significance level of *p* < 0.05. Bar: 5 µm. Magnification: 1000×. Stained perimeter: *** C≠I-0H (*p* = 0.0005), **** C≠I-24H (*p* < 0.0001), ** C≠I-4D (*p* = 0.0092), **** I-0H≠I-4D (*p* < 0.0001), **** I-0H≠I-7D (*p* < 0.0001), **** I-24H≠I-4D (*p* < 0.0001), **** I-24H≠I-7D (*p* < 0.0001); Stained area: ** C≠I-24H (*p* = 0.0068), *** C≠I-4D (*p* = 0.0001), **** C≠I-7D (*p* < 0.0001), * I-0H≠I-4D (*p* = 0.0202), ** I-0H≠I-7D (*p* = 0.0073); Diameter: *** C≠I-0H (*p* = 0.0003), ** C≠I-24H (*p* = 0.0012), * C≠I-4D (*p* = 0.0217), ** C≠I-7D (*p* = 0.0049); Endplate perimeter: **** C≠I-0H (*p* < 0.0001), ** C≠I-24H (*p* = 0.0055), **** C≠I-4D (*p* < 0.0001), **** C≠I-7D (*p* < 0.0001), * I-0H≠I-24H (*p* = 0.0417), **** I-0H≠I-4D (*p* < 0.0001), **** I-0H≠I-7D (*p* < 0.0001), **** I-24H≠I-4D (*p* < 0.0001), **** I-24H≠I-7D (*p* < 0.0001); Endplate area: *** C≠I-24H (*p* = 0.0001), * C≠I-7D (*p* = 0.0178), ** I-0H≠I-24H (*p* = 0.0067); Dispersion: *** C≠I-0H (*p* = 0.0005), ** C≠I-24H (*p* = 0.0043), ** C≠I-4D (*p* = 0.0014), * C≠I-7D (*p* = 0.0119).

The results for the stained perimeter of synaptophysin indicate an increase of 43.54% in I-0H (*p* = 0.0005) and 61.27% in I-24H (*p* < 0.0001) and a reduction of 35.81% in I-4D (*p* = 0.0092) and 29.1% in I-7D compared to C (Figure 2A). The stained area, in turn, was 18.87%, 50.72%, 64.87%, and 69.90% larger in I-0H, I-24H (*p* = 0.0068), I-4D (*p* = 0.0001), and I-7D (*p* < 0.0001), respectively (Figure 2B). The diameter was also larger in all groups, with an increase of 18.87% in I-0H (*p* = 0.0003), 50.72% in I-24H (*p* = 0.0012), 64.87% in I-4D (*p* = 0.0217), and 69.8% in I-7D (*p* = 0.0049) (Figure 2C).

The total perimeter was 60.48% and 33.10% larger in I-0H (*p* < 0.0001) and I-24H (*p* = 0.0055) and 44.6% smaller and 51.92% smaller in I-4D (*p* < 0.0001) and I-7D (*p* < 0.0001), respectively (Figure 2D). The total area was 12.47% larger in I-0H, 58.79% larger in I-24H (*p* = 0.0001), 36.98% larger in I-4D, and 42.49% larger in I-7D (*p* = 0.0178) (Figure 2E). The dispersion was 23.72%, 20.43%, 22.17%, and 19.05% larger in I-0H (*p* = 0.0005), I-24H (*p* = 0.0043), I-4D (*p* = 0.0014), and I-7D (*p* = 0.0119), respectively (Figure 2F).

### 3.3. Calcium Channels

**Figure 3:**
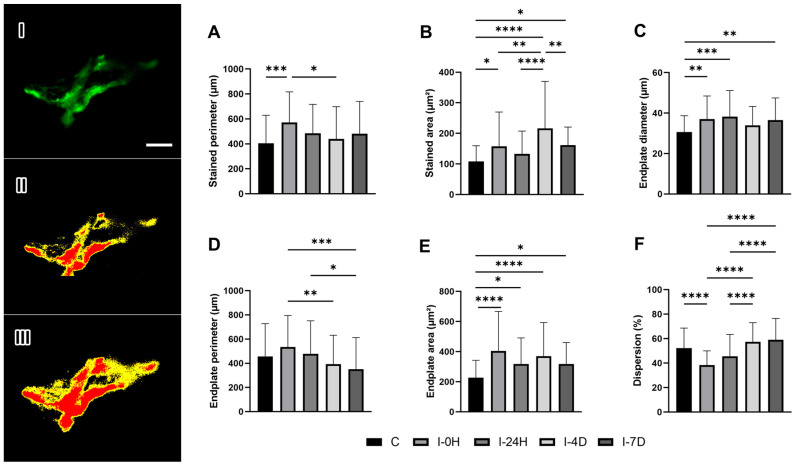
Immunofluorescence microscopy of calcium channels (green staining) in the presynaptic region (**I**) and measurement of stained area and perimeter (**II**) and total area and perimeter (**III**). Means and standard deviations of (**A**) stained perimeter (µm), (**B**) stained area (µm^2^), (**C**) endplate diameter (µm), (**D**) endplate perimeter (µm), (**E**) endplate area (µm^2^), and (**F**) dispersion (%) of experimental groups (*n* = 5/group). Data statistically treated using one-way ANOVA analysis with Bonferroni post-test with a significance level of *p* < 0.05. Bar: 5 µm. Magnification: 1000×. Stained perimeter: *** C≠I-0H (*p* = 0.0004), * I-0H≠I-4D (*p* = 0.0104); Stained area: * C≠I-0H (*p* = 0.0243), **** C≠I-4D (*p* < 0.0001), * C≠I-7D (*p* = 0.0149), **** I-24H≠I-4D (*p* < 0.0001), ** I-4D≠I-7D (*p* = 0.0099); Diameter: ** C≠I-0H (*p* = 0.0029), *** C≠I-24H (*p* = 0.0002), ** C≠I-7D (*p* = 0.0074); Endplate perimeter: ** I-0H≠I-4D (*p* = 0.0094), *** I-0H≠I-7D (*p* = 0.0002), * I-24H≠I-7D (*p* = 0.0303); Endplate area: **** C≠I-0H (*p* < 0.0001),* C≠I-24H (*p* = 0.0420), **** C≠I-4D (*p* < 0.0001), * C≠I-7D (*p* = 0.0395); Dispersion: **** C≠I-0H (*p* < 0.0001), **** I-0H≠I-4D (*p* < 0.0001), **** I-0H≠I-7D (*p* < 0.0001), **** I-24H≠I-4D (*p* < 0.0001), **** I-24H≠I-7D (*p* < 0.0001).

The stained perimeter data increased by 40.87% in I-0H (*p* = 0.0004), 19.74% in I-24H, 8.45% in I-4D, and 18.33% in I-7D (Figure 3A). The stained area increased by 45.65%, 22.64%, 100.18%, and 104.43% in I-0H (*p* = 0.0243), I-24H, I-4D (*p* < 0.0001), and I-7D (*p* = 0.0149), respectively (Figure 3B). The diameter also increased in all injured groups, with an increase of 20.83% in I-0H (*p* = 0.0029), 24.91% in I-24H (*p* = 0.0002), 10.94% in I-4D, and 16.36% in I-7D (*p* = 0.0074) compared to C (Figure 3C).

The total perimeter, in turn, increased by 17.12% and 4.7% in I-0H and I-24H, while it decreased by 13.97% in I-4D and 23.11% in I-7D, without significant results compared to C (Figure 3D). The total area results were 77.89%, 39.86%, 62.59%, and 40% larger in I-0H (*p* < 0.0001), I-24H (*p* = 0.0420), I-4D (*p* < 0.0001), and I-7D (*p* = 0.0395), respectively (Figure 3E). Calcium channel dispersion was 26.01% smaller in I-0H, 12.59% smaller in I-24H, 10.15% larger in I-4D, and 13.37% larger in I-7D (*p* < 0.0001) (Figure 3F).

### 3.4. Postsynaptic Region: α-Bungarotoxin

**Figure 4:**
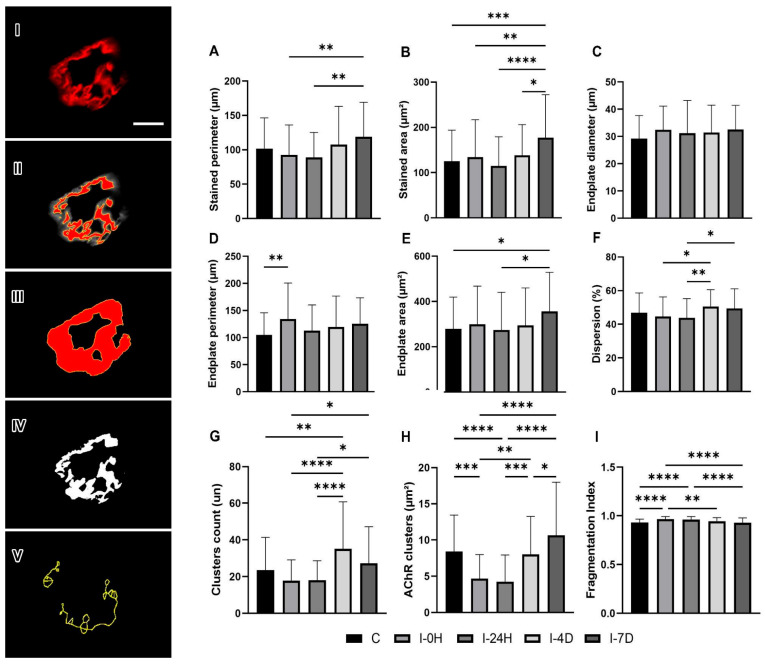
Immunofluorescence (red staining) microscopy of α-bungarotoxin in the postsynaptic region (**I**) and measurement of stained area and perimeter (**II**), area and perimeter (**III**), binarization (**IV**), and cluster count (**V**). Means and standard deviations of (**A**) stained perimeter (µm), (**B**) stained area (µm^2^), (**C**) endplate diameter (µm), (**D**) endplate perimeter (µm), (**E**) endplatearea (µm^2^), (**F**) dispersion (%), (**G**) clusters count (un), (**H**) ACHR clusters area (µm^2^) and (**I**) fragmentation (0–2) of all experimental groups (*n* = 5/group). Data statistically treated using one-way ANOVA analysis with Bonferroni post-hoc test with a significance level of *p* < 0.05. Bar: 5 µm. Magnification: 1000×. Stained perimeter: ** I-0H≠I-7D (*p* = 0.0070), ** I-24H≠I-7D (*p* = 0.0012); Stained area: *** C≠I-7D (*p* = 0.0004), ** I-0H≠I-7D (*p* = 0.0073), **** I-24H≠I-7D (*p* < 0.0001), * I-4D≠I-7D (*p* = 0.0199); Endplate perimeter: ** C≠I-0H (*p* = 0.0092); Endplate area: * C≠I-7D (*p* = 0.0405), * I-24H≠I-7D (*p* = 0.0253); Dispersion: * I-0H≠I-4D (*p* = 0.0151), ** I-24H≠I-4D (*p* = 0.0033), * I-4D≠I-7D (*p* = 0.0281); Clusters Count: **** C≠I-0H (*p* < 0.0001), * C≠I-24H (*p* = 0.0257), ** I-0H≠I-4D (*p* = 0.0014), **** I-0H≠I-7D (*p* < 0.0001), * I-4D≠I-7D (*p* = 0.0261); ACHR clusters:: *** C≠I-0H (*p* = 0.0002), **** C≠I-24H (*p* < 0.0001), ** I-0H≠I-4D (*p* = 0.0015), **** I-0H≠I-7D (*p* < 0.0001), *** I-24H≠I-4D (*p* = 0.0002), **** I-24H≠I-7D (*p* < 0.0001), * I-4D≠I-7D (*p* = 0.00236); Fragmentation: **** C≠I-0H (*p* < 0.0001), **** C≠I-24H (*p* < 0.0001), ** I-0H≠I-4D (*p* = 0.0062), **** I-0H≠I-7D (*p* < 0.0001), **** I-24H≠I-7D (*p* < 0.0001).

The stained perimeter of the postsynaptic region was 9.08% and 12.79% smaller in I-0H and I-24H and 5.69% and 16.79% larger in I-4D and I-7D, respectively, but without significant results in relation to C (Figure 4A). Meanwhile, the stained area was 7.1% larger in I-0H, 8.38% smaller in I-24H, and 10.29% and 41.58% larger in I-4D and I-7D (*p* = 0.0004) (Figure 4B). The diameter also increased by 11.08% in I-0H, 6.66% in I-24H, 7.52% in I-4D, and 11.14% in I-7D, but did not show significant results (Figure 4C).

The results for total perimeter increased by 27.81%, 7.52%, 14%, and 19.61% in I-0H (*p* = 0.0092), I-24H, I-4D, and I-7D (Figure 4D). The total area, in turn, was 1.72% smaller in I-24H, and larger by 7.39%, 5.45%, and 27.68% in I-0H, I-4D, and I-7D (*p* = 0.0405), respectively (Figure 4E). While the dispersion of the postsynaptic region decreased by 4.67% and 6.38% in I-0H and I-24H, it increased by 7.97% and 5.55% in I-4D and I-7D, respectively (Figure 4F).

The number of clusters per junction increased by 94.89% in I-0H (*p* < 0.0001), 51.3% in I-24H (*p* = 0.0257), and 30.59% in I-4D, which decreased by 37.42% in I-7D (Figure 4G), and its area was 44.28% smaller in I-0H (*p* = 0.0002), 49.4% smaller in I-24H (*p* < 0.0001), 4.76% smaller in I-4D, and 26.78% larger in I-7D compared to C (Figure 4H). Finally, fragmentation increased by 3.22%, 3.44%, and 1.5% in I-0H (*p* < 0.0001), I-24H (*p* < 0.0001), and I-4D, respectively, and decreased by 46.23% in I-7D (Figure 4I).

## 4. Discussion

Our study evaluated the morphological and histochemical changes in the triceps brachii muscle and the structure of its neuromuscular junctions in response to eccentric exercise. In the first few hours, significant modifications were observed, including an increase in cross-sectional area and density of type II muscle fibers. Over time (I-24H), a progressive reorganization was observed in the pre- and post-synaptic regions, demonstrating the neuromuscular adaptive capacity in response to the injury induced by the protocol.

Our findings indicated higher CSA levels in the injured groups, primarily in fast-twitch (Type II) fibers, which were more pronounced in group I-7D. Through these results, glycolytic predominance was observed in both groups, as expected due to the physiological characteristics already described in relation to the triceps brachii muscle [24,25], characterized by the force-velocity relationship dominated by the presence of Type II fibers.

However, due to the protocol period performed (2 h and 6 min duration), the I-0H group presented higher density and CSA of Type I fibers; this fiber type decreased after 24 h. The phenomenon of fiber type predominance is triggered in the myosin chain of the fiber, enabling the transition from slow to fast fibers, as evidenced by studies such as those by Pette and Staron (2000) and Douglas (2017) [26,27].

According to the study by Singh et al. [28], individuals who lead a sedentary life can trigger a series of comorbidities, including obesity, a disease characterized by the accumulation of adipose tissue and inflammation, which promotes a reduction in the cross-sectional area and the number of nuclei and capillaries per muscle fiber, in addition to inducing an increase in connective tissue, which has repercussions in adaptations at the NMJ.

The significant changes observed in the composition of muscle fibers suggest the beginning of an atrophy process in the triceps brachii. In response to injury, both myofibers and motor neurons reactivate cellular development programs [29,30], which, combined with morphological changes and alterations in NMJ transmission, compromise excitation-contraction coupling and result in a decrease in contractile capacity [31].

These findings highlight the progressive degeneration of the neuromuscular system and the consequent dysfunction in synaptic communication of the motor unit. Previous studies have also demonstrated that fast-twitch motor units are more vulnerable to degeneration compared to slow-twitch units [32,33], which corroborates the results presented here.

The triceps brachii muscle has great potential for strength and work capacity because of its muscle volume, and is therefore considered the strongest muscle in the elbow joint, its main function being to extend the elbow [34].

We observed an increase in both the stained area and the total area of synaptophysin in the lesioned groups. These findings corroborate those of Alder et al. [35], in which synaptophysin overexpression increases the frequency of spontaneous acetylcholine release in motor neurons, while its absence produces the opposite effect, showing that this protein plays an extremely important role in neuronal transmission.

Furthermore, the reduction in synaptophysin perimeter, associated with an increase in its dispersion, suggests an adaptive process of this protein in response to muscle injury. This reorganization may reflect adjustments in the availability and positioning of synaptic vesicles, ensuring the maintenance of neuromuscular transmission efficiency during the tissue repair process, as observed in the study by Liu et al. [36].

In fast synapses, exocytosis is controlled by the entry of calcium ions. The cytoplasmic fragment of synaptophysin has a calcium-binding site and, for this reason, may be directly involved in the calcium-dependent firing mechanism, responsible for initiating the fusion of the vesicular membrane with the presynaptic membrane and, consequently, the release of the neurotransmitter into the synaptic cleft, as observed in the studies by Thiel [37]; Kolos, Grigoriyev and Korzhevskyi [5]; and Deschenes et al. [21].

Calcium channels are characterized as high-voltage channels and play a fundamental role in the exocytosis process of acetylcholine (ACh) vesicles at synaptic terminals [38]. In this study, calcium channels showed an increase in area, perimeter, and diameter immediately after the protocol (I-0H). However, 24 h after the lesion, their stained and total perimeter began to return to the conformational pattern, which demonstrates a high capacity for reorganization of these channels.

The stained and total areas showed similar behavior to that observed for the perimeter up to the fourth day after the protocol (I-4D). However, in the I-7D group, a new increase in these variables was observed, indicating that the muscle was still in the repair process, without achieving full structural recovery. This characteristic ensures the maintenance of the functionality of the channels, an essential factor for the adaptive responses of the neuromuscular system to constant stimuli, since it regulates the influx and efflux of calcium in the process of transmitting nerve impulses from the axonal terminal to the sarcolemma of the muscle fiber [7,39,40].

According to our study, there was a smaller area and perimeter in the first hours after the protocol (I-0H and I-24H), with a return to C levels from the fourth day onwards. According to Krause Neto et al. [41], exercise protocols in adult rats triggered adaptations in the area and perimeter of the postsynaptic region due to its tissue plasticity.

One of these adaptations is related to the compact effect, where AChR receptors occupy a larger proportion of the total area of the endplate, resulting in less dispersion [42], in addition to the adaptation of the perimeter and area occupied by AChR of postsynaptic vesicles, which promotes efficiency in communication in this region and prolonged muscle fatigue and stimulates an increase in the diameter of the neuromuscular junction, even if it is not statistically significant [43,44].

Our findings showed a higher fragmentation index in the I-0H and I-24H groups, a variable that indicates the redistribution of presynaptic components over a larger synaptic area [45,46]. This is due to impaired neuromuscular transmission, that is, it affects the performance of synapses linked to nerve terminal growth [31,47].

It is possible that such findings may also be related to muscle fatigue, which, although late, may be related to the appearance of neuronal lesions or motor neuron death leading to denervation of muscle fibers followed by improved reinnervation by a new neuronal sprout from neighboring neurons [48,49]. Studies show that NMJ fragmentation can occur before the onset of sarcopenia in older adults [50], representing a physiological and progressive deterioration of the homeostatic processes of the entire organism, broadly defined as the time-dependent functional decline that affects most living organisms [51].

Although the present study focused on the morphological and histochemical characterization of the neuromuscular junction and muscle fibers following eccentric contraction-induced injury, functional assessments were performed in a complementary study derived from the same research [52]. In that study, parameters such as muscle performance were evaluated to investigate the functional consequences associated with eccentric contraction-induced injury. Therefore, the current manuscript was specifically designed to provide a detailed structural analysis, contributing to a broader understanding of the neuromuscular adaptations when considered together with the previously reported functional findings.

## 5. Conclusions

We concluded that the eccentric contraction protocol promotes significant morphological and histochemical changes in the triceps brachii muscle, through increased CSA and density, with a predominance of Type II fibers and maintenance of Type I fibers, demonstrating the effectiveness of the protocol in terms of disrupting the structure of the muscle tissue. At the NMJ, we observed an increase in variables related to individual aspects of the pre- and post-synaptic receptor clefts associated with an increase in the distribution of synaptophysin and calcium channels, in addition to the reorganization of AChR clusters and their fragmentation, which indicates the compaction of its structure and corroborates the maintenance of integrated density and the number of junctions as a positive response to the repair and adaptation mechanisms provided by the eccentric contraction protocol.

## Data Availability

The original contributions presented in this study are included in the article. Further inquiries can be directed to the corresponding author.

## Abbreviations

The following abbreviations are used in this manuscript:

NMJ	Neuromuscular Junction
CSA	Cross-sectional area
I	Injury
C	Control
NGS	Normal Goat Serum
